# Examining teachers’ behavioural intention for online teaching after COVID-19 pandemic: A large-scale survey

**DOI:** 10.1007/s10639-022-11417-6

**Published:** 2022-11-07

**Authors:** Hang Khong, Ismail Celik, Tinh T. T. Le, Van Thi Thanh Lai, Andy Nguyen, Hong Bui

**Affiliations:** 1 Faculty of Education, Monash University, Melbourne, Australia; 2 Learning & Educational Technology Research Unit (LET), University of Oulu, Oulu, Finland; 3 University of Science and Education, The University of Danang, Danang, Vietnam; 4 Birmingham City University, Birmingham, UK; 5 International School, Vietnam National University Hanoi, Hanoi, Vietnam

**Keywords:** Online teaching, Innovativeness, COVID-19, Technology Acceptance Model (TAM), TPACK, K-12 education

## Abstract

Recently, the coronavirus disease 2019 (COVID-19) pandemic has led to rapid digitalisation in education, requiring educators to adopt several technologies simultaneously for online learning and teaching. Using a large-scale survey (*N* = 1740), this study aims to construct a model that predicts teachers’ extensive technology acceptance by extending the Technology Acceptance Model (TAM) with their technological pedagogical content knowledge (TPACK) and innovativeness. TAM has been a valuable tool to measure the adoption of new technology in various contexts, including education. However, TAM has been designed and principally applied to assess user acceptance of a specific technology implementation. This study has extended TAM to measure teachers’ technology-enabled practice (online teaching) with the adoption of various technologies. The proposed model explains teachers’ behavioural intention to teach online with a good fit. Our findings revealed the collective effects of TPACK, perceived usefulness (PU) of technology, and innovativeness on teachers’ behavioural intention to teach online post-pandemic. Moreover, the study identified training and support from school as a significant predictor for both teachers’ TPACK and PU. The novelty of this study lies in its model conceptualisation that incorporates both information-technology-based constructs and personal-competence-based features, including TPACK and innovativeness. Furthermore, our study contributes to the growing body of literature that addresses the online teaching adoption by schoolteachers in the post-pandemic era.

## Introduction

Over the past decades, digital technologies have transformed the landscape of education, enabling educational innovations to transform teaching and learning activities (Luckin et al., 2012). With the affordances of technology, teachers can now have more options in terms of delivery modes (e.g., online and blended, in addition to face-to-face). At the same time, learners can participate in online learning environments beyond the boundary of a particular physical space. More importantly, the presence of electronic resources in classroom settings leads to a number of changes in educational contents such as instruction, assessment, classroom management, as well as classroom interactions. Digital tools and devices contribute to enabling students’ expanded, diverse and enriched learning experiences (Boticki et al., 2015; Domingo & Garganté, 2016; Koh et al., 2017) both inside and outside classroom boundaries, although the impact level varies among different regions, countries, schools, and teachers. Furthermore, this development provides learners with more learning opportunities and flexibility in their studies regardless of their locations compared to traditional face-to-face mode, thereby meeting the needs of diverse students. However, technology-enhanced learning and teaching seem more prevalent in higher education than in K-12 settings prior to the recent COVID-19 pandemic (Chou & Chou, 2021).

Indeed, the COVID-19 pandemic has highlighted the role of technology-enhanced learning and teaching across all educational levels and resulted in the adoption of online teaching among K-12 schools on a global scale (Adedoyin & Soykan, 2020). The health crisis caused harsh restrictions and lockdowns in various parts of the world, forcing massive school closures and interrupting the education of more than 1.5 billion students (UNESCO, 2022). According to UNESCO (2022), as of 16th September 2021, about 117 million students were still out of school. The unprecedented closures have prompted millions of teachers and students worldwide to switch from face-to-face to online or hybrid learning overnight. The pandemic has made online teaching a norm in formal education in many countries since it is a must to provide continuous education to most students (Scherer et al., 2021).

The sudden switch to the online mode posed multiple challenges to many countries worldwide (Bergdahl & Nouri, 2021; Kovacs et al., 2021; Pham et al., 2021). It required teachers to swiftly equip themselves with digital competence to cope with the new mode(s) of delivery. In developing countries such as Vietnam, the context of this study, the practice of online teaching was even more challenging due to a number of policy, human resources, and infrastructure constraints (Le et al., 2022a, b). Teachers had to adapt to unfamiliar ways of teaching and new modes of delivery in which they had no prior experience (Schlichter, 2020). As the pandemic still has no certain end in sight, the future of education is uncertain. It is argued that educational technology can support agile learning and teaching models and enhance learning performance in the post-pandemic era. As noted by Leask and Younie (2022), technology has great potential to enable us to “do education differently” (p. 188) both during the pandemic and post-COVID-19.

A question still remains if teachers have the intention to continue the online mode of education after the pandemic subsides. Although the online mode may be reverted to the face-to-face one when the COVID-19 situation improves, it has a vital role in the future of education. Furthermore, with rapid changes in educational technology, new teaching and learning modes may emerge in the coming years, rendering the importance of predicting educators’ technological behaviour. Together, it is critical to gain a deep understanding of what facilitates educators’ acceptance of online teaching in the pandemic context to inform practice in similar emergencies and to cope with uncertainties that may happen.

The Technology Acceptance Model (TAM) has been proven to be a helpful tool to measure teachers’ technology adoption by explaining their behavioural intentions (Hong et al., 2021; Nikou & Economides, 2019; Pynoo et al., 2012; Scherer et al., 2019; Teo, 2011, 2019; Wong, 2016). In other words, when explaining teachers’ adoption of technology, the TAM model stands out as one of the most popular models being validated extensively across different educational contexts. However, there is a need to further test the generalisation of TAM for online teaching acceptance beyond examining a specific system integration. At the same time, TAM has been subject to critique for not including domain-specific factors in educational contexts (Legris et al., 2003). Previous research has suggested that a linkage to teachers’ digital competencies and knowledge could effectively address the shortcomings of TAM and enhance understanding of technology acceptance processes (Scherer et al., 2019). Regarding teachers’ knowledge required for a successful integration or adoption of educational technology, the Technological Pedagogical Content Knowledge (TPACK) framework has served as a comprehensive instrument for assessment (Celik, 2022; Yeh et al., 2021). In the same vein, we argue that TPACK could also complement TAM in predicting technology adoption at a broader level, as seen in online teaching.

Nevertheless, a systematic understanding of how teachers’ perceived knowledge contributes to their technology and online teaching acceptance seems still missing. There have been few attempts to investigate the association between TPACK and TAM for teachers’ adoption of technology (Joo et al., 2018; Mei et al., 2018). For example, Li (2021) examined factors indicating Chinese teachers’ online teaching readiness in the pandemic by measuring two main constructs of TAM and three TPACK constructs. However, the study only covers English-as-a-foreign-language teachers who may be more technologically advanced thanks to their English proficiency but does not target their behavioural intention. Thus, it is crucial to draw on both TAM and TPACK to investigate teachers’ intention to continue the online mode of delivery post-pandemic when education is strongly digitalised. In addition, although educators’ innovative mindset is found to positively influence their technology adoption (Liu et al., 2010), there is little understanding of the relationship between educators’ innovative mindset and their intention to teach online. Therefore, to address the gaps above, this study aims to extend TAM with TPACK to examine schoolteachers’ continuance intention to teach online, particularly in developing countries in the post-COVID-19 era. This study seeks to answer the following research questions:

- RQ1. To what extent do teachers’ perceived usefulness, attitude, training and support, resources and infrastructure associate with their continuance intention to teach online?
- RQ2. How does teachers’ TPACK influence their continuance intention to teach online?
- RQ3. How does teachers’ innovativeness influence their continuance intention to teach online?

To answer these research questions, a large-scale cross-sessional survey was conducted with secondary school teachers in Vietnam. Structural equation modelling was applied to examine the relationships among the variables and to explain teachers' intention to teach online after the pandemic. Our findings shed light on the integration of TPACK into TAM to measure the general behavioural intention to use digital technology in education. Furthermore, this study offers unique insights into the technology adoption in Vietnam’s K-12 education as a case of developing countries in the COVID-19 pandemic.

## Theoretical background

### Technology Acceptance Model

TAM was proposed by Davis (1986) and Davis et al. (1989) based on the Theory of Reasoned Action (TRA) by Azjen and Fishbein (1980). While TRA explains general human behaviour, TAM has been validated to explain factors that determine the acceptance of information systems. Perceived ease of use (PEU) and perceived usefulness (PU) are considered the key variables that directly or indirectly explain behavioural intention. PU refers to the extent to which technology would help to improve a user's performance, while PEU refers to the effort required for the user to be able to use an information system effectively (Davis, 1989). Accordingly, the easier a technology is to use, the more useful it is to users. While PEU has a direct impact on PU, the opposite may not hold true. External factors such as facilitating conditions can influence both PEU and PU. Together, PEU and PU exert direct impacts on attitude toward using the system, which, in turn, influences one’s behavioural intention to use it (BI). PU also has a direct effect on BI. Finally, BI is associated with the actual use of the system.

Since its introduction, TAM has been widely used to explain factors underlying users’ technology acceptance. In the field of education, it is predominantly adopted to predict teachers’ technology integration in their practices. The first line of research targets educators’ acceptance of a specific technology. For example, Nikou and Economides (2019) used TAM to survey European teachers’ intention to use mobile-based assessments. PU was confirmed to have a direct impact on the intention, while facilitating conditions had an indirect effect. Likewise, Pynoo et al. (2012) applied TAM to report Flemish and Dutch teachers’ acceptance and use of an educational portal called KlasCement. The most significant predictors of BI to use the portal were found to be attitude and PU. Moreover, Armenteros et al. (2013) used TAM to survey instructors' BI towards multimedia teaching materials.

The second line of literature focuses on teachers’ acceptance of technology in general or a series of technologies together. For instance, Teo (2011) tested a model combining TAM and other theories among 592 Singaporean teachers. The results showed that PU, attitude, and facilitating conditions directly influenced their future intention to integrate technology, while PU was positively related to attitude. Similarly, Teo et al. (2019) found that both the PU of Web 2.0 technologies and facilitating conditions positively influenced Chinese pre-service teachers’ intention to use them in the future. However, while the study by Wong (2016) conducted in the Hongkong context also supported the direct path from attitude and facilitating conditions to BI, it rejected the influence of PU on the intention. Despite the dominance of TAM in teachers’ technology adoption, the model has not been used widely in studies predicting teachers’ acceptance of online teaching, particularly amidst the pandemic (Chou & Chou, 2021; Sangeeta & Tandon, 2021), except Chen et al. (2021). In their study, teachers’ intention to teach online was primarily accounted for by their cognitive attitude, which was influenced by PU, thus confirming TAM hypotheses.

**Fig. 1:**
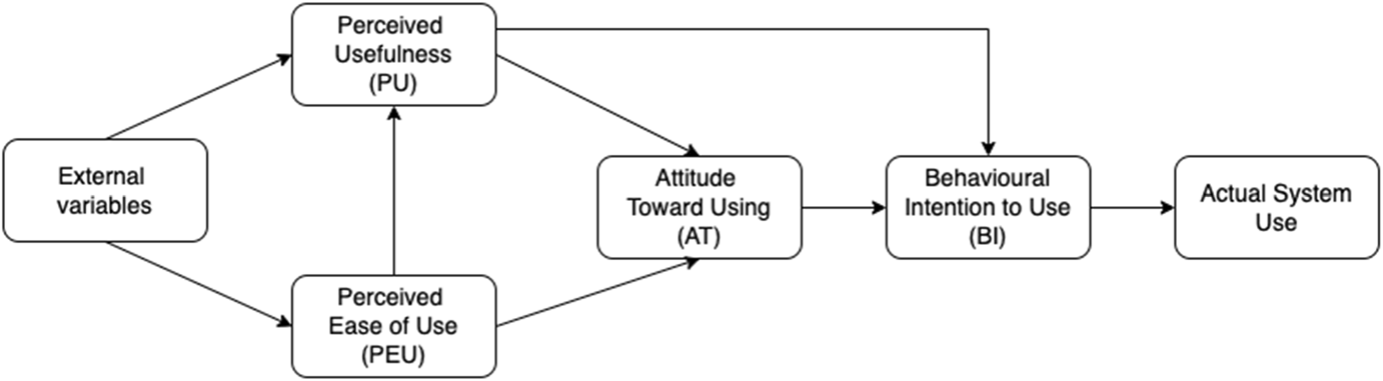
Technology Acceptance Model (TAM) (Davis et al., 1989)

While TAM can explain teachers’ technology acceptance well, the model is not without critique. For example, Koehler et al. (2014) argued that TAM is inefficient to conceptualise what it means to accept and incorporate information and communications technology (ICT) in classrooms. The model does not include domain-specific factors that can influence users in educational contexts (Legris et al., 2003). For instance, it does not cover the types of professional knowledge teachers must have to become proficient in ICT adoption in teaching and learning. Furthermore, how teachers perceive their capacity to use technology is an important factor to consider when examining teaching supported by technology (Mei et al., 2018). Teo et al. (2019) believed that teachers might apply a new technology if they perceive it as relevant to their subjects and/or specific didactical approaches. Hence, they suggested that the TPACK framework could address the shortcomings of the TAM and enhance the understanding of technology acceptance processes in classrooms. TPACK may be better than TAM in understanding how teachers make decisions to integrate ICT into teaching and learning processes. Thus, in the next session, TPACK will be discussed as a framework to explore teachers’ intention to integrate technology into their practices (Fig. 1).

### Teachers’ knowledge (TPACK) for successful educational technology integration

In order to integrate technology effectively in education, teachers are required to not only enhance their ICT capacity but also incorporate their professional knowledge with technology-enhanced practice (Damşa et al., 2021; Nguyen et al., 2021). Digital competence required for teachers involves (1) generic digital competence, which refers to generic digital skills, knowledge and attitudes required of teachers to operate in their work contexts; (2) subject-matter digital competence, which denotes their capacity to use ICT in teaching disciplinary knowledge; and (3) profession-related digital competence which entails their knowledge and skills to perform professional activities such as designing lessons in digital environments (Gudmundsdottir & Hatlevik, 2018). While the terms mentioned above may not necessarily mean the same across the studies, there is a consensus that teachers need not only the ability to use digital tools but also the ability to establish technology-enabled learning with the pedagogical knowledge to foster meaningful understanding.

TPACK (or previously known as TPCK) has been a widely adopted model to measure teachers’ competence in adopting and using technology in learning and teaching (Voogt et al., 2013; Zimmermann et al., 2021). TPACK is not the simple combination of separate knowledge sets. Rather, it is a construct consisting of seven elements including content knowledge (CK), pedagogical knowledge (PK), technology knowledge (TK), pedagogical content knowledge (PCK), technological pedagogical knowledge (TPK), and technological pedagogical content knowledge (TPCK), which exist in an intertwining relationships and interactions (Mishra & Koehler, 2006). The acronym “TPACK” (rather than TPCK) accentuates the idea of TPACK being the “Total PACKage” for effectively integrating technology into teaching (Thompson & Mishra, 2007, p. 38).

Previous studies have investigated the interrelationship between pre-service/in-service teachers’ TPACK and their beliefs regarding pedagogical issues (e.g., Niess, 2005; So & Kim, 2009) or technology use (Niess, 2005; Ozgun-Koca, 2009). Findings from these studies show both the encouraging and hindering impacts of teachers’ beliefs on the extent to which they apply technology and how they integrate technology in their teaching practice. Regarding measuring teachers’ TPACK, previous studies employed different research methods including surveys (e.g., Doering et al., 2009), interviews, observation (e.g., Chisholm & Padgett, 2004), document analysis or a mix of methods (e.g., Akyuz, 2018) to evaluate teacher’s competencies and their readiness to integrate technology in their teaching activities. Findings generally show the potential of the TPACK framework in supporting the assessment of teachers’ competencies and the prediction of their teaching quality in relation to educational technology use (Abbitt, 2011; Akyuz, 2018; Tsankov & Damyanov, 2019; Wang et al., 2018).

In addition to being an indicator of teachers’ readiness to integrate technology into their lessons, TPACK has also been used as a key dimension of teachers’ online teaching readiness construct during the COVID-19 pandemic (Howard et al., 2021; Scherer et al., 2021). This echoes well with Teo’s (2019) and Scherer et al.’s (2019) suggestion that TPACK can effectively supplement TAM in comprehending influential factors that contribute to teachers’ readiness to implement online teaching.

### Conceptual model and hypothesis development

**Fig. 2:**
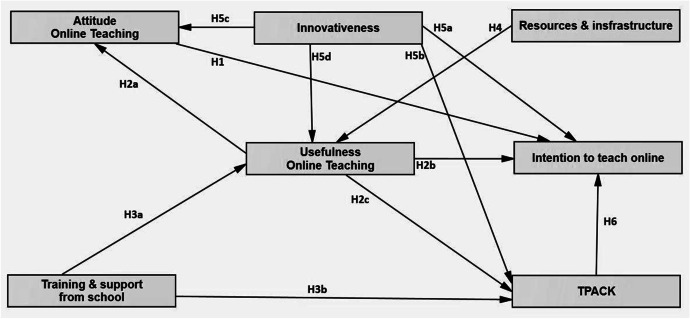
Conceptual model for the study

Based on the identified gaps, this study conceptualises a model integrating TPACK into TAM to explain teachers’ behavioural intention to teach online after the COVID-19 pandemic. Given limited literature examining teachers’ online teaching intention in emergency contexts, we build our hypotheses mostly on the studies related to both online teaching or e-learning readiness prior to and during the COVID-19 and teacher’s use of technology since online teaching requires educators to integrate various technological tools to deliver lessons effectively. This model includes seven variables of TPACK, attitude toward online teaching, perceived usefulness of online teaching, personal innovativeness, school training and support, school infrastructure and resources, and intention to teach online. Figure 2 shows the conceptual model.

#### Attitude toward behavioural intention for online teaching

Attitude is one of the core variables in the original TAM (Davis et al., 1989). Teachers’ attitude toward technology tends to have an impact on their intention to use it in teaching. This is supported in both studies focusing on pre-service teachers (Ahmet et al., 2016; Sadaf et al., 2012; Teo, 2011), and those targeting in-service ones (Nikou & Economides, 2019; Pynoo et al., 2012; Wong, 2016). Teo (2012) argued that attitude was the biggest influencing factor on pre-service teachers’ intention to adopt technology, which was corroborated by Chen et al. (2021) when exploring college teachers’ intention to teach online. Similarly, Hung and Jeng (2013) confirmed the positive relationship between the two variables. Therefore, it is hypothesised:

- *H1. Attitude (AT) toward online teaching is related to behavioural intention (BI) to teach online*.

#### Perceived usefulness of online teaching

PU refers to a person’s belief that a particular technology would make their work more efficient and it could be defined as “the degree to which a person believes that using a particular system will enhance job performance” (Davis, 1989, p. 320). In the context of education, research has evidenced a positive relationship between teachers’ perception of usefulness and their attitude toward technology as well as their intention to use technology (Scherer et al., 2019; Teo, 2011). In a study by Cigdem and Topcu (2015), teachers’ PU was the strongest predictor of their BI to use a learning management system. Likewise, if teachers form ideas that online teaching would be helpful and effective in their work, they are likely to adopt this mode of delivery. While teachers are forced to adopt online teaching during the COVID-19 pandemic, debate continues about the best strategies for effective online teaching and questions have been raised about the usefulness of online teaching in comparison with the traditional face-to-face classroom (van der Spoel et al., 2020). Nevertheless, teachers’ perception of usefulness is still an appropriate indicator for their AT towards online teaching and BI for future practice with this channel. Therefore, the following hypotheses are formed:

- *H2a. Perceived usefulness (PU) of online teaching is related to attitude (AT) toward online teaching.*
- *H2b. Perceived usefulness (PU) of online teaching is related to behavioural intention (BI) to teach online*.

In addition, teacher motivation to adopt a new practice is moderated by various factors, among which is the utility filter (Nolen et al., 2014). This essentially means that teachers choose to engage in certain ideas and practices they perceive as useful to reach a goal. For example, if they consider online teaching as effective to fulfil their work, they may make efforts to invest time in exploring new online learning platforms and tools to deliver quality instruction. Hence, we hypothesise that:

- *H2c. Perceived usefulness (PU) is related to technological pedagogical content knowledge (TPACK)*.

#### Training and support from school

To facilitate the adoption of technology, the presence of technology training and support is crucial (Venkatesh et al., 2003). In TAM, technology training and support refers to facilitating conditions, an external variable that constitutes environmental factors that support technology use (Scherer et al., 2020). Many studies have indicated a positive relationship between facilitating conditions and PEU (Nikou & Economides, 2017; Teo, 2011; Teo et al., 2019; Wong, 2016). Despite this, technology adoption in education, particularly in the context of less developed countries, has been challenging with a lack of change management support, technology support, and technology resources to ease teachers in utilising relevant e-learning tools to provide a more effective learning delivery and student experience. Providing sufficient training and support for technology adoption would help teachers better acknowledge the usefulness of educational technology as well as improve their TPACK. Therefore, the following hypotheses are proposed:

- *H3a. Training and support (TS) is related to perceived usefulness (PU).*
- *H3b. Training and support (TS) is related to technological pedagogical content knowledge (TPACK).*

#### Resources and infrastructure

Similar to technical training and support, resources and infrastructure also play an important role in teachers’ acceptance of technology integration and form part of facilitating conditions (Venkatesh et al., 2003). In the case of online and blended learning, teachers not only need good devices but also reliable internet connection, easy-to-use learning management system, and quality teaching and learning resources (Mohee & Perris, 2021). The availability of resources and infrastructure can influence teachers’ perception of whether it is possible and effective to conduct online lessons, and thus how useful this new mode of delivery is. The following hypothesis is proposed:

- *H4. Resources and infrastructure (RI) are related to the perceived usefulness (PU) of online teaching*.

#### Personal innovativeness

Personal innovativeness (PI) is a construct related to technology acceptance proposed by Agarwal and Prasad (1998). Accordingly, a person is considered innovative if they are willing to experiment with new technology. Personal innovativeness has been documented in the literature as having positive correlations with PEU (Nikou & Economides, 2017) and intention to use technology (Agarwal & Prasad, 1998; Crespo & Rodríguez, 2008; Liu et al., 2010). PI can be expanded to denote a mindset of being willing to try new things in general and having high tolerance for uncertainty. In educational contexts, innovative teachers embrace, initiate, and model changes (Powell et al., 2014). They want to try and lead others to implement new pedagogical approaches, assessment methods, technologies, and new modes of delivery, which are all important when they need to switch from face-to-face to online teaching or blended learning. When educators take risks to adopt innovative technologies in their daily lessons, it is expected that their TPACK would be enhanced. Thus, we propose the following hypotheses.

- *H5a. Personal innovativeness (PI) is related to behavioural intention (BI) to teach online.*
- *H5b. Personal innovativeness (PI) is related to TPACK.*
- *H5c. Personal innovativeness (PI) is related to attitude (AT) toward online teaching.*
- *H5d. Personal innovativeness (PI) is related to perceived usefulness (PU).*

#### Technological Pedagogical Content Knowledge (TPACK)

Due to lack of necessary knowledge about pedagogy to give online lessons, many teachers fail to effectively teach their subject in online learning environments (McAllister & Graham, 2016). To accomplish online lessons, it is crucial for teachers to understand the pedagogical aspect of online learning technologies (Howard et al., 2021). For instance, Zoom software offers breakout rooms for participants during an online meeting. When a teacher plans teamwork activities or group discussions in a Zoom lesson, they should have the knowledge to use breakout rooms. Such practices are closely related to TPK. Similarly, teachers often need domain-specific TK in online teaching (Lachner et al., 2021). Accordingly, a mathematics teacher most probably uses different applications compared to a geography counterpart in their online lesson. Thus, teachers also should be aware of and have the TK specific to their teaching field. Teachers are required to have diverse knowledge and skills to initiate an effective online teaching process (Gudmundsdottir & Hathaway, 2020). These knowledge domains cover both technical knowledge (e.g., scheduling a lesson, using online teaching software) and pedagogical knowledge (e.g., giving timely and adaptive feedback) (Scherer et al., 2021). Additionally, TPACK is crucial for teachers to answer students’ questions to overcome their misunderstandings during online teaching by using appropriate technology (Benson & Ward, 2013). In light of this, the below hypothesis is proposed:

- *H6. Technological pedagogical content knowledge (TPACK) is related to behavioural intention (BI) to teach online*

## Methodology

To investigate teachers’ intention to continue online teaching post-pandemic, a large-scale cross-sessional survey among Vietnamese secondary school teachers was conducted in a community project to support local schools in their digital transformation to respond to the disruption caused by the deadly COVID-19’s fourth wave sweeping across the country.

### Participants and procedures

Data collection involved a large-scale cross-sectional survey associated with a policy consulting project for a local government in Vietnam. Participants for this study included secondary school teachers (N_male_ = 668; N_female_ = 1070). In developing countries like Vietnam, the practice of online or blended learning was largely absent in schools prior to the pandemic. Thus, emergency online education during the health crisis poses multiple challenges for teachers, including issues related to curriculum, pedagogy, assessment in an online environment, student access, infrastructure, and technical support (Khlaif et al., 2021). Particularly, online teaching stresses the need for teachers to effectively use various tools and applications for both pedagogical and professional purposes.

A convenient sampling strategy was adopted via the anonymous online survey offered by Qualtrics to encourage participants to share openly. Data collection took place in November 2021. An introduction letter was sent to the Department of Education and Training of the Province (DET) to introduce the project with support from the local council head. Then, an invitation with the online survey hyperlink was sent to all secondary school teachers via internal contact emails of DET. Participants were asked to complete all the questions, which took less than ten minutes. The demographic data of the participants can be found in Table 1.

**Table 1:** The demographic profile of participants

Measures	Items	Frequency (*N* = 1740)	Percentage
Gender	Male	668	38.4
	Female	1070	61.5
	Not revealed	2	0.1
School location	Urban area	613	35.2
	Rural area^a^	819	47.1
	Mountainous area	308	17.7
School level	Middle school	811	46.6
	High school	825	47.4
	Multi-level school	104	6.0

^a^In this study, a rural area is a geographic area that is located outside towns and cities but in the plain; meanwhile, mountainous areas refer to the geographic areas covered by mountains with a higher than average percentage of ethnic minority groups

### Measures

To capture teachers’ intention for online teaching after the pandemic, we adapted the items from existing surveys. In detail, attitude toward online teaching items were adapted from An et al. (2021). Training and support from school, and resources and infrastructure were adapted from Mohee and Perris (2021) for blended learning. Personal innovativeness items were adapted from Powell et al. (2014), while TPACK was adapted from Howard et al. (2021). Behavioural intention was adapted from Mei et al. (2018), whereas perceived usefulness items were adapted from Stockless (2017). There are 41 items across seven constructs (see Appendix for details). A seven-point Likert-type scale was used to measure all items with 1 corresponding to “strongly disagree” and 7 to “strongly agree”.

The questionnaire was initially constructed in English and then translated into Vietnamese, the native language of the surveyed teachers. The committee approach, back-translation and pre-test procedure by Sperber et al. (1994) were strictly followed. The translation was made by two researchers of the team, who are fluent in both languages and then further checked by a linguistic expert to ensure linguistic equivalence. Ten people assisted in the pre-test procedure to ensure that the questionnaire was of the highest level of translation and understanding. Internal consistency of the instruments (i.e., Cronbach's alpha values) is presented in Table 2.

**Table 2:** Research instruments (survey items included in Appendix)

Sources	Measures	Cronbach’s alpha
An et al. (2021)	Attitude (AT) towards online teaching	0.92
Mei et al. (2018)	Behavioural intention (BI)	0.75
Stockless (2017)	Perceived usefulness (PU)	0.75
Mohee and Perris (2021)	Training and support	0.96
	Resources and infrastructure	0.87
Powell et al. (2014)	Personal innovativeness	0.95
Howard et al. (2021)	Technological Pedagogical Content Knowledge (TPACK)	0.95

### Data analysis

In this study, we performed the structural equation modelling (SEM) analysis to discover the existing relationships among seven variables of the hypothesised research model. SEM analysis is an analytical approach to reveal the causal associations among multiple variables (Schumacker & Lomax, 2004). In this study, the predicting relationships among the constructs of TAM (perceived usefulness, attitude toward online teaching, intention for online teaching), TPACK framework and innovative behaviour were investigated by maximum likelihood estimation based on SEM approach. Also, the research model incorporated resources and infrastructure, and support from school. In the research model, an equation was calculated by endogenous (dependent) and exogenous (independent) variables. The hypothesized model consisted of three endogenous (innovativeness, resource and infrastructure, training from support) and four exogenous variables (TPACK, attitude, perceived usefulness, and behavioural intention). Both direct and indirect associations of exogenous variables with endogenous variables were estimated. We checked the required assumptions before the SEM analysis. For the normality assumption, we found skewness and kurtosis coefficients acceptable. Therefore, no bootstrapping was performed. Besides, no outliers and missing data were observed. The estimated equations are reported by the path coefficients, namely the standardized regression weights (betas). Statistical analyses were performed by means of SPSS (Statistical Package for Social Sciences) 22.0 and AMOS (Analysis of Moment Structures) 18.0 software.

## Results

As demonstrated in Table 3, behavioural intention is positively correlated with TPACK component and usefulness at a moderate level. Similarly, perceived usefulness was moderately associated with TPACK and intention. Furthermore, there was also a positive correlation between usefulness and innovativeness. Also, both training and support, and resources and infrastructure were positively related to intention. However, attitude is weakly and positively correlated with innovativeness and intention. Training and support are positively related to resources and infrastructure at a high level.

**Table 3:** Bivariate correlations among the research variables

	2	3	4	5	6	7
Training & support (1)	0.735**	0.383**	0.491**	-0.024	0.358**	0.491**
Resources & infrastructure (2)		0.415**	0.463**	-0.038	0.338**	0.474**
Usefulness (3)			0.492**	-0.026	0.545**	0.355**
TPACK (4)				0.012	0.589**	0.668**
Attitude (5)					0.062*	0.056*
Intention (6)						0.542**
Innovativeness (7)						

**. Correlation is significant at the 0.01 level (2-tailed)

*. Correlation is significant at the 0.05 level (2-tailed)

The structural equation analysis is conducted to test the relationships among the research variables: perceived usefulness, training and support from school, resources and infrastructure, personal innovativeness, TPACK, intention for online teaching, and attitude toward online teaching. After removing the insignificant relations from the hypothesised model, the research model is acceptable with the results indicating a robust fit: χ2/df = 2.26; GFI = 0.998; AGFI = 0.990; CFI = 0.999; TLI = 0.995; NFI = 0.998; RMSEA = 0.027 (according to good and acceptable fit indices suggested by Hu and Bentler (1999).

**Fig. 3:**
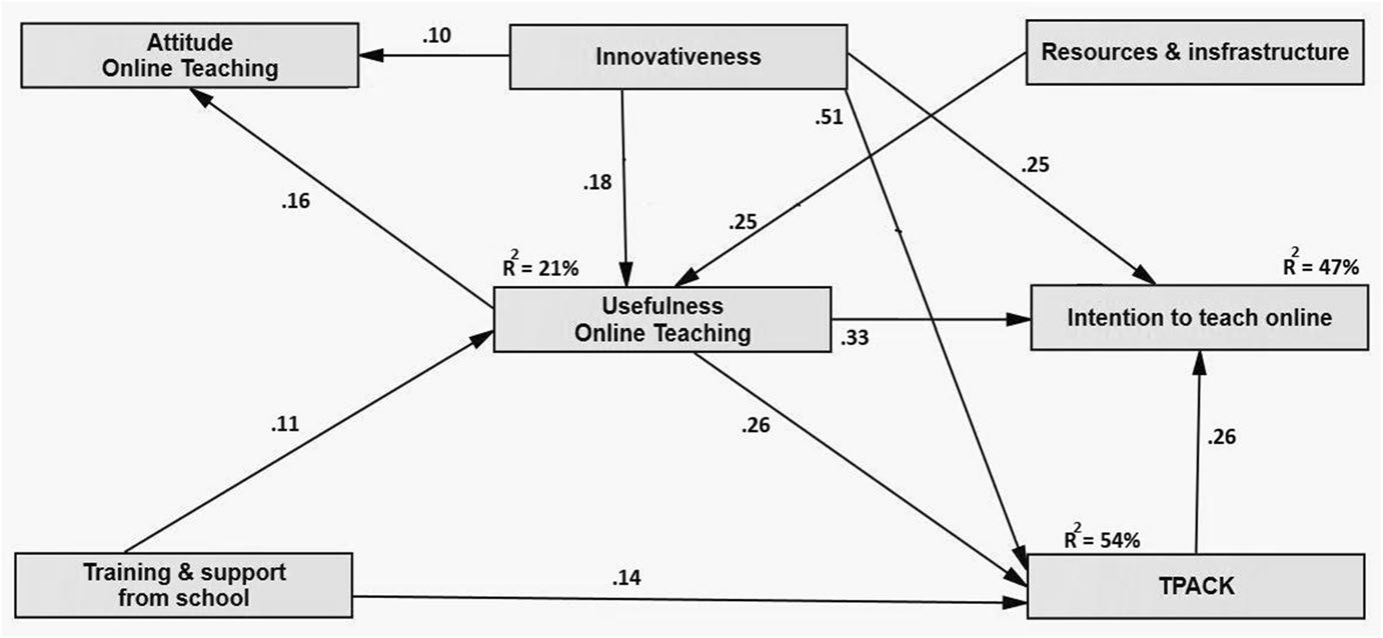
The research model with standardised estimates

As depicted in Fig. 3, training and support from school are found to positively affect the perceived usefulness of online teaching (*β* = 0.11; H3a accepted) and technological pedagogical content knowledge (*β* = 0.14; H3b accepted). Moreover, according to the research model, the usefulness of online teaching has a positive effect on attitude toward online teaching (*β* = 0.16; H2a accepted), technological pedagogical content knowledge (*β* = 0.26; H2c accepted) and intention to teach online (*β* = 0.33; H2b accepted). However, the attitude toward online teaching (p > 0.05; H1 rejected) has no significant relationship with the intention to teach online. In addition, a positive effect of TPACK (*β* = 0.26; H6 accepted) on the intention to teach online was observed.

The research model reveals that innovativeness is positively associated with attitude online teaching (*β* = 0.10; H5c accepted), usefulness online teaching (*β* = 0.18; H5d accepted) and intention to teach online (*β* = 0.25; H5a accepted). Furthermore, innovativeness has a positive effect on TPACK (*β* = 0.51; H5b accepted). A positive relation between resources and infrastructure and the usefulness of online teaching is also found (*β* = 0.25; H4 accepted).

The usefulness of online teaching and TPACK combined with innovativeness explain 47% of intentions to teach online. Together, training and support from school, resources and infrastructure, and innovativeness explain 21% of the usefulness of online teaching. In aggregate, the combined effects of the usefulness of online teaching, innovativeness, and training and support from schools account for 54% of the variance in TPACK.

## Discussion

This study has examined factors that impact schoolteachers’ intention to teach online post-pandemic, using a less developed region in Vietnam as a case for developing countries. Drawing on both TAM and TPACK frameworks, the study investigates the teachers’ perceived usefulness of online teaching, attitude towards this mode of delivery, training and support, resources and infrastructure, TPACK, innovative mindset, and their behavioural intention to conduct online lessons. It shows that the proposed conceptual model works well in this context when ten in eleven hypotheses are confirmed. In the following sections, these hypotheses are discussed with regard to each research question.

- *RQ1. To what extent do perceived usefulness, attitude, training and support, resources and infrastructure associate with teachers' continuance intention to teach online?*

SEM analysis yielded a positive relationship between perceived usefulness and attitude toward online teaching (H2a). In other words, the more teachers perceive teaching in online environments as beneficial, the more positive their attitude towards online teaching will be. This finding is in line with TAM while similar results were reported in previous studies about teachers’ technology adoption (Joo et al., 2018; Khlaif et al., 2022; Scherer et al., 2019; Teo, 2011).

Similarly, the findings show that teachers’ perceived usefulness of online teaching is positively related to their intention to deliver online classes (H2b). That is, those teachers who recognise online teaching as beneficial for their courses are more likely to choose the online teaching mode. A positive association between perceived usefulness and behavioural intention in educators’ integration of technology is also observed elsewhere (Cigdem & Topcu, 2015; Nikou & Economides, 2017; Pynoo et al., 2012; Teo, 2011). The findings from this current study suggest that it is important to pay attention to how teachers think about online education. A positive experience with online courses as learners and observation of effective online educators may help them appreciate the strengths of this delivery mode and be willing to practise it.

Our model supports the hypothesis that teachers’ perceived usefulness regarding online teaching could lead to an increase in their TPACK (H2c). This finding implies that teachers can better assess suitable online teaching technologies for their domain when they believe that online teaching is effective. This could be explained from a psychological lens of utility filters (Nolen et al., 2014). As long as teachers perceive a practice or type of knowledge as helpful to achieve their goals, they will engage themselves in that practice or activities to gain the knowledge. As online teaching requires the use of multiple digital tools, teachers are likely to make efforts to gain knowledge of those tools if they consider them as useful. The finding resonates with previous research (Li, 2021), which revealed a positive association of teachers’ utility perceptions about ICT tools with their TPACK.

Surprisingly and contrary to the hypothesis based on TAM, teachers’ attitude toward online teaching does not predict their online teaching intentions (H1). Previous studies have reported mixed results regarding the association between attitude and intention. For example, whereas Blackwell et al. (2016), Chen et al. (2021), Hung and Jeng (2013), Khlaif et al. (2022) and Teo (2011) show a positive relationship, others report contradictory findings (Venkatesh, 2000). In this current study, the lack of association between the two variables may be due to the compulsory nature of online learning during the pandemic and the top-down decision-making model characterised by centralised education systems like the one in Vietnam. No matter whether teachers liked online teaching or not, online lessons were mandated in some parts of the country for certain periods of time amidst the pandemic.

The current literature shows that facilitating conditions or contextual factors can positively influence educators’ technology adoption (Ameen et al., 2019; Mei et al., 2018; Nikou & Economides, 2017; Teo, 2011; Teo et al., 2019; Wong, 2016) and their TPACK (Porras-Hernández & Salinas-Amescua, 2013). The results of this current study confirmed that both training and support from school (H3a) as well as resources and infrastructure (H4) positively influence perceived usefulness of online teaching. This finding is similar in developed countries, like Germany, with a positive influence of quality of teacher training during the COVID-19 pandemic on teachers’ use of digital learning materials in future teaching (Paetsch & Drechsel, 2021). Online teaching, particularly in the pandemic context, requires practitioners to handle a number of digital tools and resources within a short notice. Therefore, the pedagogical and technical support by their schools would ease the burden and help them deliver online lessons more effectively. Such support can facilitate the new mode as a useful, urgent and needed alternative to the face-to-face mode. Moreover, the online teaching mode might fail without the availability of educational digital materials and adequate facilities, a challenge that Vietnam is facing amidst the pandemic. Consequently, the lack of support and resources would negatively impact the feasibility and quality of online teaching, thereby impacting educators’ perception of its usefulness.

- *RQ2. How does teachers’ TPACK influence their continuance intention to teach online?*

The core component of the TPACK framework is explored in this study since it represents both the technological pedagogical and technological content-related aspects of online teaching (Howard et al., 2021). Educators in general and online educators in particular need to go beyond simply using digital tools but exploiting them to provide students with quality learning (Ertmer & Ottenbreit-Leftwich, 2013; Kabakci Yurdakul et al., 2012). This necessitates their capacity to integrate content, pedagogy and technology effectively (Koehler & Mishra, 2009). This study demonstrated that a high level of TPACK can enhance teachers’ teaching intention in online mode (H6). In other words, teachers with more knowledge in their domain-specific online teaching technologies are more likely to deliver online lessons more eagerly. Similarly, the combined knowledge in all content, pedagogy and technology allows teachers to effectively instruct online. Existing studies also confirmed a predicting relationship between TPACK and adoption of educational technology, such as Chinese pre-service English teachers with stronger TPACK being more likely to accept computer-assisted language learning 2.0 (Mei et al., 2018). In fact, TPACK has been used as an important variable to measure teachers’ online teaching readiness during the COVID-19 pandemic (Howard et al., 2021; Scherer et al., 2021). In addition, other studies of teachers amidst the pandemic demonstrated a positive influence of online teaching self-efficacy, a scale closely related to TPACK scales used in this study, and their intention to integrate technology in their practice (Chou & Chou, 2021; Menabò et al., 2021). Therefore, it is argued that teachers’ TPACK efficacy can be a leading indicator of their continuous intention to work in virtual environments and there exists a strong need to enhance their TPACK for online or distance education. In the context of Vietnam, the need even becomes stronger as only about one third of teachers often use digital tools for teaching and managing students online (Le et al., 2022a). The findings suggest that professional development activities that aim to prepare teachers for online teaching should focus on not only pedagogical aspects of technologies but also content knowledge. It is also crucial for online teaching software developers to consider different domain representations (e.g., maths and chemistry) for customising content knowledge.

- *RQ3. How does teachers’ innovativeness influence their continuance intention to teach online?*

The level of personal innovativeness is regarded as an important factor contributing to teachers’ adoption of technology or innovation (Rogers, 2003). The results confirm that innovative teachers are more likely to continue to teach online (H5a), which echoes existing research related to teachers’ use of technology (Agarwal & Prasad, 1998; Crespo & Rodríguez, 2008; Liu et al., 2010). Moreover, personal innovativeness also predicted teachers’ positive attitudes towards online teaching (H5c), perceived usefulness (H5d), and TPACK (H5b). According to these findings, innovative teachers with a positive online teaching attitude tend to appreciate the educational values of online education. Furthermore, such teachers might be more knowledgeable in selecting appropriate online teaching technologies for their teaching field. For many teachers, particularly in the context of Vietnam, giving lessons in an online environment was a brand-new experience during the pandemic. Hence, their innovativeness played an important role in contributing to their online teaching intention. A rapid transition from the face-to-face to the online mode induced by the COVID-19 pandemic, resulting in the lack of adequate training, also forced teachers to continuously experiment with new online platforms, applications and tools for teaching and learning, as well as exploring electronic resources to perform their work effectively. Innovative educators who are willing to try new technology (Agarwal & Prasad, 1998) would embrace the changes and take initiative to find solutions to online teaching challenges more than less innovative colleagues. As a result, they would learn more about technology, hold a more positive attitude, find online teaching more useful, and have a greater willingness to continue the online mode of delivery. In a school environment where new ideas about technology are encouraged, practised, and shared, educators may be more accepting of the changes or risks induced by online teaching. In this aspect, the Vietnamese education system still largely appreciates educators who follow the sequence and content established in the textbook or by the authority, thereby discouraging them to diverge from the norm.

Although online education had been offered at both K-12 and higher education settings prior to the COVID-19 pandemic, it was certainly not adopted on a massive scale as observed during the crisis. Contrary to voluntary adoption in the past, online delivery mode was made compulsory in many countries to cope with rapid social distancing and school closures during the pandemic. Furthermore, even when a completely online delivery may subside as the pandemic situation improves, it is possible that some forms of online education may remain, particularly for the benefit of children with special needs and disabilities. Given agile teaching and learning modes in the future, it is critical to predict educators’ technological behaviour from online teaching during the pandemic in order to inform future policy, research, and practice. However, despite the existence of investigations into online teaching intention amidst the pandemic (Chen et al., 2021; Chou & Chou, 2021; Sangeeta & Tandon, 2021), a deeper and more comprehensive understanding of factors influencing their continuation to deliver online lessons post-pandemic is needed. This study contributes to that growing body of literature.

This study extends TAM with TPACK to examine user intention by integrating their constructs for a technology-enabled practice with the adoption of various technologies. Accordingly, the conceptualised model was implemented to examine educators’ behavioural intention to online teaching beyond the pandemic. TAM is considered a well-known model for explaining teachers’ integration of technology (Hong et al., 2021; Nikou & Economides, 2017, 2019; Scherer et al., 2019; Teo, 2011, 2019; Wong, 2016) and to some extent online teaching (Chen et al., 2021), while TPACK has also been used as an indicator for the integration (Ifinedo et al., 2020) and online teaching readiness (Çınar et al., 2021; Howard et al., 2021; Scherer et al., 2021). There are few studies to date that combine both TAM and TPACK to explore influencing factors on teachers’ adoption of technology (Joo et al., 2018; Mei et al., 2018). In the context of the pandemic, Li (2021) explored Chinese teachers’ online teaching readiness by measuring two main constructs of TAM and three TPACK constructs. However, it is not clear how TAM and TPACK can be combined to investigate teachers’ intention to continue the online mode of delivery after the pandemic until this current study. We find that perceived usefulness and TPACK were associated with educators’ continuance. Thus, TPACK construct can be used to extend TAM to predict teachers’ continuance intention. Surprisingly, the findings reveal that attitudes toward online teaching may not demonstrate a significant relationship with intention for online teaching. Where this result is not in line with TAM, it could be possibly explained by the mandatory nature of emergency remote teaching. Moreover, this study shows that teachers’ innovative mindset is likely to predict their online teaching intention, a construct hardly measured in existing online teaching scholarships. This finding suggests that a certain mindset can influence teachers’ technology-related behaviour.

## Limitations and future directions

Although this is a large-scale study with careful design, it cannot avoid limitations; and its results should be interpreted with caution. First, the study sample consisted of secondary school teachers in a less developed province in Vietnam. Therefore, the findings may not be generalised to those working at primary schools or higher education providers or to other wealthier provinces in the country. Second, since convenience sampling was chosen and data were collected using a cross-sectional survey, the study may not yield data which can explain causal links or other factors not investigated in this paper. Third, the use of a self-reported approach (i.e., the participants self-completed the survey items) may render social desirability bias (Jensen, 2020). For example, teachers may rate themselves higher than the actuality on some scales as they believe it would be more desirable despite the survey being anonymous. Fourth, the administration of an online survey may limit the access to those teachers who are familiar with filling online forms or better at technologies, particularly in the context of Vietnam, even though many teachers already had experience with online teaching at data collection time.

Future studies may expand the sample to include teachers at other educational levels such as primary and higher education, and other parts of the country/the world so that comparisons could be made across different groups (e.g., primary vs. secondary school teachers, public vs. private schools, urban vs. rural areas, dominant ethic vs. ethnic minority groups, developed vs. developing countries). Furthermore, while our study only explored teachers’ innovative mindset, school support, plus resources and infrastructure, other teacher factors and school factors such as belief and collegiality as well as contextual factors at student and system levels could be analysed as they may influence teachers’ continuous intention to teach online. Moreover, in this study, behavioural intention was explained by three variables at 47%, leaving 53% unaccounted for. Future investigations may consider other possible reasons to be included in the model. Finally, more research is needed to further test the power of the combined TAM and TPACK in predicting educators’ intention toward online teaching.

## Conclusion

This study is among few studies that investigated the continuance intention of teachers in a developing country to teach online after the COVID-19 pandemic using a large-scale cross-sessional survey design. Furthermore, this study seeks to address the shortcomings of TAM by involving education-related factors from TAM and innovativeness. We provide evidence that TPACK can be a useful construct to complement TAM in assessing behavioural intention for a technology-enabled practice with the adoption of various technologies in the education context. Our results demonstrate the effectiveness of the proposed model in predicting educators’ acceptance of online teaching practice in the post-pandemic era. Moreover, this study reports a positive relationship between teachers’ innovative mindset and their online teaching intention, an association not commonly investigated in the current literature about online teaching. Our findings indicate that teacher characteristics can influence their technology-related intention. This study sheds a light on future studies investigating the role of personal characteristics in technology adoption. These findings suggest several courses of action for policy, research, and practice regarding teaching and learning modes in the coming years after the COVID-19 pandemic. A key policy priority should therefore be to plan for enhancing teachers’ knowledge (i.e., TPACK, their perception of technology) and providing necessary training and support. Finally, beyond examining user adoption of a specific technology in an implementation, this research has thrown up many questions in need of further investigation on the adoption of technology-enabled practice in education.

### Implications

This study explored the role of TAM, TPACK, and innovativeness in Vietnamese teachers’ continuance behaviour towards teaching online. The findings show that facilitating conditions including professional training and technical support, digital teaching/learning resources and infrastructure (e.g., digital tools and devices, Internet, online learning platform) have positive relationships with teachers’ perceived usefulness of online teaching and TPACK, which, in turn, influence their intention to teach online in the future. These have implications at both national and school levels for Vietnam and other developing countries.

First, nationwide, it is critical for the government and Ministry of Education and Training to invest in reliable internet connections, digital devices, online teaching platforms, and resources for both teachers and students to teach and learn online effectively. A national teaching and learning resource bank such as Student Learning Space (Singapore Ministry of Education, n.d.) would be necessary to provide educators and students with an effective platform and quality educational materials. Furthermore, although Vietnamese teachers have been trained to conduct online lessons, they need much more support, particularly in terms of using online teaching tools and managing students (Le et al., 2022a). A web page with online tutorials on using the tools and other aspects of online teaching would be helpful in addition to short online or face-to-face training sessions already conducted. To promote teachers’ professional development, an online Facebook group could be helpful for teachers across the nation to share ideas for online teaching (Tay et al., 2021). In addition, to help Vietnamese teachers appreciate the online mode of delivery, teachers themselves need to have positive experience as participants in online courses. Therefore, it is expected that the online professional development programs for teachers in Vietnam (such as the ETEP program) should be well implemented in practice.

At the organisational level, school leaders are advised to survey teachers’ needs and hold additional training sessions on online teaching. They can tap on the expertise of information technology or maths teachers to provide ongoing technical support for other colleagues and facilitate internal professional learning sessions. Likewise, having an online group chat among small groups of teachers enables them to support each other with technical issues and share materials (Tay et al., 2021). Furthermore, school leaders can form professional learning communities among teachers where they can observe and discuss colleagues’ online practices frequently. Importantly, an innovation climate within a school can encourage teachers to take risks, try out innovative practices and instructional ICT innovative ideas (Chou et al., 2019). Together, all these measures would enable teachers to solve practical challenges associated with online teaching during the pandemic, thereafter, developing their TPACK and innovativeness, as well as helping them view online teaching more positively and making them more willing to teach online in the future.

## Data Availability

The dataset for the current study may be available upon request to the team.

## Appendix. Survey items

### Resources & infrastructure

1. My school has a stable internet connection which allows fast material upload and download. Infrastructure and facilities are adequate for on-site and on-campus learning and teaching activities (e.g., library, tutor office hours).
2. Infrastructure and facilities are adequate for online teaching and learning activities (e.g., computer, tablet, projector/big monitor, LMS, internal email…).
3. My school has e-learning materials and resources for online teaching and learning.
4. My school uses learning apps and websites to enhance student learning in certain subjects (e.g., hoc247.net, Khan Academy…)

### Training and support from school

5. My school has organised many training sessions on online learning platforms (e.g., Zoom, Microsoft Teams, Viettel Study, Google Meet…)
6. The training in the online learning platform provided by my school is effective.
7. My school has organised many training sessions on online teaching tools (e.g., Kahoot, Quizizz, Jamboard…)
8. The training in online teaching tools provided by my school is effective.
9. My school assigns particular staff to provide technical support for online teaching.
10. The technical support provided by my school is effective.
11. My school has guides (e.g., materials, videos…) to support teachers in online teaching.
12. The guides provided by my school are effective.

### Personal innovativeness

13. All my teaching strategies are student-centred.
14. I take initiative to learn from my colleagues.
15. I create learning environments that are flexible and allow students to develop their best selves.
16. I have an inquisitive spirit, and possess a drive for change, creativity, and imagination.
17. I have motivations for my personal development.
18. I model a growth orientation towards learning for students.
19. I embrace change.
20. I am one of those who take the lead in modelling changes for other teachers.
21. I proactively initiate change in response to students’ needs and progress.
22. I embrace trial and error as part of the process to improve teaching and learning practices.
23. I encourage other teachers to be independent and self-directed learners to upgrade their level.
24. I demonstrate the professional responsibility to contribute to the effectiveness, self-renewal, and sustainability of the teaching profession, as well as to the school and community.

### Technological pedagogical content knowledge

25. I can create an effective and interesting online lesson for my students.
26. I can use technology in the assessment of certain topics.
27. I can use student assessment to modify instruction in an online environment.
28. I can use technology to create effective online teaching contents that depart from textbook knowledge.
29. I can do my job well in an online environment.

### Perceived usefulness of online teaching

30. I believe that the current online learning is effective.
31. I believe that online teaching can be as effective as face-to-face learning.
32. I believe that online learning can change the way teachers and students think in a positive way.
33. I believe that online learning can bring a new horizon of knowledge to students.

### Online teaching intention

34. I will only teach online if my school requests me to do so.
35. I will combine online and face-to-face teaching whenever it is possible to do so.
36. I am willing to support my colleagues in online teaching.
37. I am willing to teach online even when it is not compulsory at my school.

### Attitude toward online teaching

38. I am comfortable with teaching online.
39. Online teaching is stressful.
40. I do not like teaching online.
41. In general, I am not satisfied with my online teaching in the past two years.

## Declarations

This study was approved by the Monash University Human Research Ethics Committee (no. 30676) and the research incorporated ethical principles involving informed consent and guidelines according to the Monash University Human Research Ethics Committee (MUHREC).
